# Mass Spectrometry–Based Proteomics in Clinical Diagnosis of Amyloidosis and Multiple Myeloma: A Review (2012–2024)

**DOI:** 10.1002/jms.5116

**Published:** 2025-02-19

**Authors:** Katerina Kratka, Pavel Sistik, Ivana Olivkova, Pavlina Kusnierova, Zdenek Svagera, David Stejskal

**Affiliations:** ^1^ Institute of Laboratory Medicine, Faculty of Medicine University of Ostrava Ostrava Czech Republic; ^2^ Institute of Laboratory Medicine University Hospital Ostrava Ostrava Czech Republic; ^3^ Department of Clinical Pharmacology, Institute of Laboratory Medicine University Hospital Ostrava Ostrava Czech Republic; ^4^ Department of Clinical Biochemistry University Hospital Ostrava Ostrava Czech Republic

**Keywords:** amyloidosis, liquid chromatography, mass spectrometry, multiple myeloma, proteomics

## Abstract

Proteomics is nowadays increasingly becoming part of the routine clinical practice of diagnostic laboratories, especially due to the advent of advanced mass spectrometry techniques. This review focuses on the application of proteomic analysis in the identification of pathological conditions in a hospital setting, with a particular focus on the analysis of protein biomarkers. In particular, the main purpose of the review is to highlight the challenges associated with the identification of specific disease‐causing proteins, given their complex nature and the variety of posttranslational modifications (PTMs) they can undergo. PTMs, such as phosphorylation and glycosylation, play critical roles in protein function but can also lead to diseases if dysregulated. Proteomics plays an important role especially in various medical fields ranging from cardiology, internal medicine to hemato‐oncology emphasizing the interdisciplinary nature of this field. Traditional methods such as electrophoretic or immunochemical methods have been mainstay in protein detection; however, these techniques are limited in terms of specificity and sensitivity. Examples include the diagnosis of multiple myeloma and the detection of its specific protein or amyloidosis, which relies heavily on these conventional methods, which sometimes lead to false positives or inadequate disease monitoring. Mass spectrometry in this respect emerges as a superior alternative, providing high sensitivity and specificity in the detection and quantification of specific protein sequences. This technique is particularly beneficial for monitoring minimal residual disease (MRD) in the diagnosis of multiple myeloma where traditional methods fall short. Furthermore mass spectrometry can provide precise typing of amyloid proteins, which is crucial for the appropriate treatment of amyloidosis. This review summarizes the opportunities for proteomic determination using mass spectrometry between 2012 and 2024, highlighting the transformative potential of mass spectrometry in clinical proteomics and encouraging its wider use in diagnostic laboratories.

## 1 Introduction

Early detection and identification of pathological conditions associated with protein disorders in patients in the hospital setting represents a new trend in the diagnosis of various diseases. However, the identification of pathological biomarkers, that is, specific disease‐causing proteins, is extremely difficult, time consuming and costly.

Proteins are not only biomarkers of these diseases, but their misfolded forms are often themselves the cause of pathological problems [1]. For example, amyloidosis, which is caused by the accumulation of misfolded proteins in various organ tissues, may be influenced by posttranslational modifications (PTMs). However, it is not definitively proven that PTMs are required for a protein sequence to form amyloids. Other diseases that can be caused by PTMs include Alzheimer's disease, Parkinson's disease, or cystic fibrosis.

Protein pathology can be caused by PTMs, which involve changes in the amino acid side chains of proteins. There are more than 400 types of these modifications, the most common being phosphorylation, acetylation, N‐glycosylation, amidation, and many others [2, 3]. Although these modifications are essential for the proper functioning of proteins, their disruption or excessive PTMs can lead to protein misfolding and aggregation, causing the diseases mentioned above [1, 3].

Proteomics in the hospital setting is a multidisciplinary field that overlaps significantly with various medical specialties. Cardiac surgery departments, which provide heart samples, internal medicine departments, which provide kidney or adipose tissue samples, and hemato‐oncology departments, which specialise in blood sampling, collaborate in the diagnosis of disease. The development of modern analytical methods such as mass spectrometry (MS) techniques is taking the importance of proteomics in hospitals to a new level.

Routine techniques for the determination of specific proteins of given diseases are mainly immunochemical or electrophoretic methods, but the use of these methods can be challenging in the diagnosis of some diseases. A typical example is multiple myeloma; this disease is characterized by proliferation of plasma cells in the bone marrow, leading to overproduction of nonfunctional immunoglobulins. The analyte of interest here is M‐protein (myeloma protein), a key biomarker of MM. Determination of this protein is particularly important to quantify the protein in the blood, which reflects progression and helps to monitor treatment. In addition, monitoring of minimal residual disease (MRD) is useful. Routinely, is this protein determined from blood using electrophoretic methods? Although electrophoresis is a sensitive method, significant interferences can occur in this case. This is due to interference with monoclonal antibodies used in the treatment of MM, which can lead to false positive results [4]. Despite the great advantages that electrophoretic methods provide, they cannot fully cover the need for MRD monitoring.

The diagnosis of amyloidosis can also be mentioned. As previously stated, it is a disease in which misfolded proteins are deposited in tissues, resulting in organ dysfunction. Detection of this disease is performed at pathology institutes by Congo red staining and observation of green plaques under polarized light, which is the gold standard for routine diagnosis of this disease. This type of detection can be further supplemented by immunohistochemical (IHC) examination of the exact type of amyloid, but this additional determination may not be completely specific [5], and only 76% of amyloid types are correctly identified by IHC. However, accurate identification of the specific type of amyloidosis is critical for making treatment decisions and predicting disease progression.

Approximately 30 amyloid proteins have been documented in the literature. However, according to the article by Dasari et al. [6], out of nearly 16 000 human tissue and fat samples, 58.99% were identified as the immunoglobulin light chain type (AL). Amyloid transthyretin (ATTR) was the second most common, with an incidence of 28.44%. Published data suggest that these two types account for 90% of all amyloidosis cases. Table 1 lists the clinical parameters of the most frequent amyloid proteins, as well as parameters related to myeloid M‐protein.

**TABLE 1:** The most frequently occurring amyloid proteins and multiple myeloid M‐protein and their clinical parameters [7, 8].

Protein	Nomenclature	Molecular weight (MW, Da)	Associated diseases	Clinical significance	Structure of amyloid	Primary organs affected
Immunoglobulin light chains	AL	25 000	Primary amyloidosis	Monoclonal immunoglobulins form amyloid deposits	Fibrillar structure	Kidneys, heart, nervous system
Transthyretin	ATTR	13 800 (homotetrameric protein 55 000)	Familial amyloid polyneuropathy, senile systemic amyloidosis	Mutations lead to incorrect amyloid conformation and deposition, causing multisystem diseases	Beta‐sheet‐rich fibrils	Heart, nervous system, liver
Monoclonal immunoglobulin	M‐protein	Variable depending on the type of immunoglobulin	Multiple myeloma	Overproduction of monoclonal immunoglobulin (M‐protein), which can cause organ damage	Normal immunoglobulin structure	Bone marrow, kidneys, bones, nervous system

Human blood contains a wide range of proteins, with a concentration of approximately 60–80 g/L. In the plasma, which is the liquid part of the blood, about 60% of the total protein content is albumin, 35% consists of various types of globulins, and the remainder is fibrinogen. Overall, plasma contains around 10 000 different proteins, with concentrations spanning a broad range of about 12 orders of magnitude. If amyloid proteins proliferate in the tissues, they are also washed into the bloodstream, where the concentration of amyloid protein can in some cases be as high as 1 g/L in the case of AL or 0.1 g/L in the case of serum amyloidosis [7]. The concentration of proteins such as transthyretin (TTR) does not increase dramatically during ATTR amyloidosis. In this type, mutation and formation of amyloid fibrils predominantly occur, rather than its overproduction. In this regard, we are able to detect its presence; however, we are unable to determine the site of occurrence in the tissues.

In the context of pathological conditions associated with protein deposition, simple, specific and sensitive techniques for their determination are becoming increasingly important. In routine clinical diagnostics in healthcare facilities, MS is gaining importance because it can not only identify but also quantify the individual structural forms of these proteins with minimal interference compared to the previously used analytical techniques. The present review provides a comprehensive overview of clinically important proteins analyzed by MS in clinical laboratories published between 2012 and 2024.

This review describes clinically relevant pathological proteins in different biological matrices. Proteomic approaches for the identification and quantification of amyloid and multiple myeloma using liquid chromatography combined with MS detection are discussed.

## 2 Introduction to Proteomic MS

MS has become an indispensable tool in modern proteomics, providing high sensitivity, specificity and detailed structural information about proteins. Its applications extend far beyond proteomic analysis alone, making it a cornerstone technology in several areas of biological and clinical research.

The widespread use of MS in proteomics is primarily due to its exceptional sensitivity and specificity, allowing the detection and identification of proteins at very low concentrations, making it invaluable in proteomics and clinical research This capability is enhanced by advanced technologies like tandem mass spectrometry (MS/MS) [9, 10]. This is particularly important in clinical diagnostics, such as the measurement of m‐protein concentrations in blood for the diagnosis of multiple myeloma and the monitoring of MRD [11]. In addition, MS is a powerful tool for structural analysis of proteins in proteomics due to its ability to distinguish molecules based on their mass‐to‐charge ratio (m/z) and fragmentation patterns (MSn). Fragmentation provides unique information about protein structure, including amino acid sequence. Ion mobility spectrometry (IMS) adds another dimension by analyzing ion conformation via collision cross section (CCS), which helps distinguish isobaric and conformationally different proteins. Isotopic resolution improves the accuracy of molecular weight determination and can distinguish between isotopic forms of proteins. Spectral libraries allow protein identification by comparing experimental spectra with known reference data. Techniques such as electron capture dissociation (ECD) and electron transfer dissociation (ETD) preserve PTMs during fragmentation, making them critical for the analysis of large proteins and modified forms. Native mass spectrometry (Native MS) is used to study proteins in their native state, revealing protein–protein and protein–lipid interactions and providing insight into quaternary structures and functional complexes. Together, these advanced techniques provide a comprehensive understanding of protein structure and function in biological systems [12, 13].A key advantage of MS is its ability to simultaneously identify and quantify proteins in complex biological mixtures. This capability is essential for large‐scale proteomic studies where thousands of proteins need to be analyzed and their abundances compared across samples [14, 15].

Compared to traditional protein measurement methods used in clinical biochemistry laboratories, such as electrophoretic and immunochemical techniques, MS offers several advantages. Electrophoretic methods have limited sensitivity for low abundance proteins and suffer from low reproducibility. Immunochemical methods, while highly sensitive and specific (e.g., ELISA), have limited multiplexing capabilities, require specific antibodies for each target protein and cannot measure PTMs [16, 17, 18]. MS offers several unique capabilities not offered by traditional methods. It allows the simultaneous analysis of thousands of proteins, facilitating complex studies of proteomic changes in biological systems. MS also allows detailed characterization of PTMs, which is essential for understanding the regulatory mechanisms of protein function [14, 19]. In addition, MS can achieve highly accurate quantification using isotope‐labeled standards, providing absolute concentrations of proteins and peptides in samples [20]. Another significant advantage is the ability to identify novel proteins and peptides without the need for specific antibodies, which is critical for the discovery of new biomarkers and therapeutic targets.

However, proteomic analysis using MS faces challenges, particularly with regard to the variability of patient proteomes. Diseases such as multiple myeloma exhibit significant heterogeneity, meaning that each patient sample has a unique protein profile that can be further complicated by mutations. This variability complicates the standardization of MS‐based methods, requiring an individualized approach to sample analysis and a focus on absolute quantification for accurate diagnosis [21]. In contrast, amyloid measurements do not involve such extensive variability, allowing for better standardization of methods, although accurate identification of amyloid type remains critical.

Despite these challenges, MS has become an essential tool in modern proteomics. Its ability to provide detailed structural information and highly sensitive and accurate analyses of proteins and other biomolecules makes it invaluable for advanced proteomic studies, clinical diagnostics, and a wide range of other applications in biology and medicine. MS is revolutionizing the study and understanding of complex biological systems.

## 3 Biological Matrices in Proteomic Analysis of Multiple Myeloma and Amyloidosis

### 3.1 Multiple Myeloma

In multiple myeloma, blood serum, plasma and urine are the most commonly used matrices for the detection and quantification of M‐protein, a key biochemical marker of this disease [22]. Blood serum is particularly important for the detection of M‐protein by MS, as shown in a study by Dunphy et al. [23] comparing proteomic changes in extramedullary and intramedullary myeloma. In clinical practice, serum M‐protein concentrations can range from a few mg/L to tens of grams per liter (g/L) in advanced cases. For example, serum M‐protein concentrations above 30 g/L typically indicate active multiple myeloma. A study by Chanukuppa [24] that combined serum and bone marrow analysis identified 279 and 116 differentially expressed proteins in bone marrow and serum, respectively, highlighting the importance of these matrices for diagnosis and monitoring of disease progression.

Another important sample is bone marrow, which is the primary site of tumor growth in multiple myeloma. Proteomic analysis of bone marrow allows the identification of pathological proteins and clonal plasma cells, which is critical for assessing disease progression and treatment response. M‐protein concentrations in bone marrow can be higher than in blood, depending on the number of clonal cells and the degree of bone marrow infiltration.

In addition to these traditional matrices, other biological samples have been explored that offer new possibilities for the diagnosis and monitoring of multiple myeloma. For example, saliva is being investigated as a noninvasive biofluid for detecting various diseases, including multiple myeloma. Although this is a still developing field, studies suggest that specific proteins and peptides in saliva could potentially serve as biomarkers for systemic diseases such as multiple myeloma. However, the clinical implementation of saliva‐based diagnosis of multiple myeloma is still in the research phase, and further validation is needed to develop reliable diagnostic tests for this disease using saliva samples [25].

The kidneys play an important role in the detection of M‐protein, especially in cases where multiple myeloma has caused kidney damage, known as myeloma kidney. High levels of M‐protein in the urine, known as Bence‐Jones proteinuria, can reach several hundred mg/L or more in advanced disease. The presence of M‐protein in the kidneys can lead to significant functional impairment, often detected by decreased glomerular filtration or the presence of protein in the urine.

#### 3.1.1 Amyloidosis

Tissue samples play a critical role in amyloidosis, especially biopsies of organs affected by amyloid deposition. The most commonly used tissues are adipose tissue, myocardium, kidney, liver, and peripheral nerves. These tissue samples are often formalin‐fixed and paraffin‐embedded (FFPE) for histologic and proteomic analysis [26]. FFPE blocks allow the detection and typing of amyloid, which is critical for determining appropriate treatment. Amyloid protein concentrations in these tissues can be difficult to measure due to their low levels and the presence of other abundant proteins. The article [27] describes blood levels of β‐amyloid in units of pg/mL, which makes its detection challenging.

Another potential matrix is cerebrospinal fluid (CSF), which has been studied in relation to neurological forms of amyloidosis. CSF may contain proteins and peptides related to amyloidogenesis, potentially providing new diagnostic tools for patients suspected of having central nervous system amyloidosis. However, the detection of amyloid proteins in CSF can be challenging due to their typically low concentrations, which are in the hundreds of pg/mL. In some diseases, there may also be a natural depletion, for example, in Alzhemier disease, where analyte Aβ‐42 is converted into plaques and its concentration in CSF is even more reduced [27].

The heart is another organ where amyloid proteins can accumulate, leading to cardiac amyloidosis. Amyloid protein levels in the heart can be high, especially in transthyretin amyloidosis (ATTR). Significant amounts of amyloid can be detected in heart biopsies, leading to myocardial stiffness, diastolic dysfunction, and heart failure. Quantification of amyloid proteins in the heart is typically performed by immunohistochemistry (IHC) or MS.

Although peripheral blood is a less invasive alternative to tissue biopsy, it has limited diagnostic value because it cannot determine which organ is affected by amyloidosis. For example, blood levels of amyloid proteins are often low, making them difficult to detect. For example, serum amyloid A (SAA) levels can rise to several mg/L during inflammation or amyloidosis but may be less than 10 mg/L in healthy individuals. Therefore, adipose tissue aspiration is considered an optimal solution that offers a compromise between invasiveness and diagnostic value. This approach allows the collection of a representative sample for MS analysis that can confirm the presence of amyloid and accurately determine its type, which is critical for accurate diagnosis and therapy [28, 29].

## 4 Proteomic Strategies in Amyloidosis and Multiple Myeloma

The concentration of proteins in human biological matrices varies widely depending on the type of tissue, its function, and the conditions associated with a particular disease. The complexity of these analyses is compounded by the diverse nature of biological matrices, which require meticulous preparation to ensure accurate protein extraction and minimal interference from matrix components. Because biological samples are often difficult to analyze due to matrix complexity, sample preparation is an essential part of the analytical procedure.

### 4.1 Proteomic Approaches in Amyloid Analysis

In the field of amyloid analysis, many papers focus primarily on the analysis of these proteins from organ tissues and less frequently from adipose tissue. The focus on the analysis of FFPE blocks was reported in seven articles [6, 30, 31, 32, 33, 34, 35], with the exception of the article by Dasari et al. [6], which also focuses on the analysis of adipose tissue. The analysis of adipose tissue is very complicated and therefore there are not many publications on this topic. However, the authors [6, 29, 36] were able to identify amyloid proteins even in such a complex matrix. In most cases, they identified ALκ (amyloid light kappa chain), ALλ (amyloid light lambda chain), AA (amyloid type A), and ATTR (transthyretin amyloid). There is also a focus on native tissue without fixation [37, 38]. One paper dealt with the identification of amyloid proteins from CSF [39].

The authors were able to detect different types of amyloid proteins using the laser microdissection (LMD) technique in connection with MS detection (see Table 2). In almost all articles, the authors focused only on the identification of the type of amyloidosis proteins. However, one of the papers also dealt with quantification [39]. In this article, the authors analyzed β‐amyloid‐related analytes, mainly Aβ1–38, Aβ1–40, and Aβ1–42 in CSF. The authors used isotope‐labeled internal standards of the analytes (15N51‐Aβ1–38, 15N53‐Aβ1–40, and 15N55‐Aβ1–42) to ensure accurate quantification. By using these isotope‐labeled standards, the authors were able to minimize the risks associated with sample preparation errors and largely eliminate matrix effects, thus ensuring high precision and reliability of the measurements. Accurate quantification of these peptides allows not only diagnosis but also monitoring of disease progression or treatment efficiency.

**TABLE 2:** Review of selected publications for the determination of amyloid proteins in different tissue types.

No.	Matrices	Identification Quantification	Instrumentation	Column	Analytes	Sample preparation	Software	*n*
[29]	FAT	Identification	NanoFlow‐LC	n/a	ALλ/ALκ/ATTR/AA/AGel/AIns/ALys	The sample was defatted using several steps involving soaking in acetone and air drying, then placed in 3% SDS and 0.02 M DTT, incubated at 100°C for 30 min, and then incubated overnight [37]	Mascot SEQUEST X!Tandem	75
[30]	FFPE	Identification	LC‐MS/MS	n/a	ALκ/ALλ/AA/ATTR	LMD + digestion with trypsin	Mascot SEQUEST X!Tandem	50 and 41
[31]	FFPE	Identification	NanoFlow‐LC‐MS/MS‐Dionex RSLCnano 3000 coupled to a Q‐Exactive orbitrap	Custom‐made C18 micro column	ALκ/ALλ/AA/ATTR	LMD incubation with 35 μL of 10 mM Tris + 1 mM EDTA and 0.002% Zwittergent at 98°C for 90 min, then reduced with DTT at 50°C for 30 min, alkylation with 150 mM iodoacetamide for 30 min in the dark. Acetone precipitated proteins, which were then dissolved in 20 μL of 200 mM TEAB and digested with trypsin (0.1 μg) overnight at 37°C	Mascot SEQUEST	106
[32]	FFPE	Identification	Easy NanoFlow‐LC 1000 coupled to a Q‐Exactive plus	n/a	ALκ/ALλ/AA/ATTR/FGN/ALECT 2	3‐day preparation without LMD (for more info, see [25]) Trypsin usage	Crux	121
[33]	FFPE	Identification	NanoFlow‐LC coupled to a LTQ‐Orbitrap	Optipak trap packed with Magic C8 beads + separation on 75 μm × 15 cm Magic C18 column	ALκ/ALλ/AA/ATTR	Incubation with 35 μL of 10 mM Tris + 1 mM EDTA and 0.002% Zwittergent at 98°C for 90 min, denaturation in a water bath for 60 min, digestion of 0.5 μg trypsin overnight at 37°C, reduction with 3 μL of 0.1 M DTT	Mascot SEQUEST X!Tandem	147
[34]	FFPE	Identification	Dionex Ultimate 3000 RSLC NanoFlow‐LC coupled to a Q‐Exactive Plus	Thermo Easy‐spray Acclaim PepMap column C18 (75 μm × 15 cm, 3 μm/100 Å packing)	AL/ATTR/AIns/ALECT 2/Aβ2M	LMD + proteins were extracted into 10 mM Tris/1 mm EDTA/0.002% Zwittergent buffer solution (35 μL) by heating (99°C, 1.5 h) followed by sonication (1 h) and then digested with trypsin (25 ng) overnight (~18 h) at 37°C. Sample was then reduced with DTT (50 μg) at 99°C for 5 min, freeze‐dried, reconstituted in 0.1% (v/v) trifluoroacetic acid in HPLC‐grade water (20 μL)	Mascot	640
[35]	FFPE	Identification	Orbitrap Elite Hybrid Ion Trap‐Orbitrap mass spectrometer	C18 Acclaim PepMap Nano Trap Column Separation on a 75 μm × 15 cm EASY‐Spray column C18	ALλ/ALκ/ATTR/AA	LMD + proteins were extracted into 35 μL of a 10 mM Tris/1 mM EDTA/0.002% Zwittergent buffer. Followed by heating at 98°C for 90 min. Subsequently sonicated in a water bath for 60 min and digested overnight at 37°C using 0.5 μg of trypsin. The resulting peptides were reduced using 3 μL of 0.1 M DTT at 95°C for 5 min	MaxQuant	22
[6]	FFPE FAT	Identification	Eksigent or Dionex NanoFlow‐LC coupled to a LTQ‐Velos LTQ‐Orbitrap XL Q‐Exactive‐plus	n/a	AL/ATTR/ALECT2/AA/AH/Alns/KRT5–14/Afib/AApoA4/AApoA1/AANF/Aβ2M/ASem 1/AGel/TGFBI/ALys/AIAPP/AApoC2/APro/AEnf/ACal	LMD + solubilized and digested with trypsin (FAT) Protein extraction using heat and denatured via sonication–extracted proteins were digested with trypsin (FFPE)	Mascot SEQUEST X!Tandem	16 175
[36]	FAT	Identification	NanoFlow‐LC coupled to a linear ion trap	BioBasic‐SCX column (i.d. 0.32 × 100 mm, 5 μm)/separation on BioBasic‐C18 (i.d. 0.180 × 100 mm, 5 μm)	ALκ/ALλ/AA/ATTR	Flushing with isotonic solution, maceration in 100 μL of buffer (7 M urea + 2 M thiourea + 4% CHASP + 65 mM DTT) Sonicated in cold water 4 times for 15 min, centrifuged for 1 h, followed by removal of the upper layer and dialyzed (18 h, 4°C, 3.5‐kDA membrane) against 50 mM ammonium bicarbonate. Digestion with trypsin 37°C for 2 h with the addition of trifluoroacetic acid	SEQUEST	26
[37]	Native kidney	Identification	LMD‐MS	n/a	ALκ/ALλ/AA/ ALECT2/AFib/ApoA1/ApoA4/AGel	LMD + n/a	n/a	170
[38]	Native kideny	Identification	LMD/MS	n/a	ALλ/ALκ/AH/AA/AFib/ALECT2/ApoA/AGel/Aβ2M/ATTR	LMD + proteins were extracted into 35 μL of a 10 mM Tris/1 mM EDTA/0.002% Zwittergent buffer and then digested overnight by trypsin	SEQUEST Mascot X!Tandem	127
[39]	CSF fluid	Identification Quantification	UHPLC coupled to a QTRAP 6500+	Atlantis HILIC Silica column (3 μm, 2.1 × 50 mm)	Aβ1–38/Aβ1–40/Aβ1–42	SPE extraction more in article [33]	n/a	5

Abbreviation: n/a, not available.

Regarding the samples, the largest number was analyzed in the publication [6], with a total of 16 175 samples over 10 years (2008–2018). Of these, 58.99% were identified as light chain amyloidosis and 28.44% as ATTR amyloidosis. Over the years, the authors of this study identified many other less common amyloid proteins (see Table 2), offering comprehensive insights into amyloid subtypes.

As far as sample preparation was stated, microdissected tissue from FFPE blocks was always incubated in 35 μL of 10 mM Tris + 1 mM EDTA and 0.002% Zwittergent buffer at 98°C for 90 min [6, 31, 33, 34, 35, 38] Authors of articles focusing on FFPE tissue blocks agree on this procedure. This method has the advantage of efficiently breaking down cross‐links formed during the fixation process, providing better access to proteins for downstream analysis. However, the reliance on high temperatures and chemical buffers could result in some protein degradation. The next steps vary from author to author, but the final step is always overnight trypsin digestion at 37°C, which is a standard approach for bottom‐up proteomics. Although this method provides effective protein digestion, the existence of some more resistant proteins may potentially affect the accuracy of protein quantification due to incomplete digestion. In addition, any residual cross‐linking could prevent complete degradation of proteins, especially in highly fixed tissues. In contrast, sample preparation of adipose tissue, its preparation is not very standardized, and different approaches can be used. For example, in one paper [29], the tissue was defatted in several steps including soaking in acetone and air drying. This preparation reduces lipid interference but is time consuming and can lead to protein loss. Another paper [36] described simpler approach of rinsing with isotonic solution and followed by maceration in 100 μL buffer (composition in Table 2), followed by subsequent preparation with centrifugation and dialysis steps, ending with trypsin digestion, which is also found in other articles using adipose tissue [6, 30]. Although this method was faster, this method risks incomplete removal of lipids, it may lead to problems such as ion suppression in MS and reduced detection sensitivity for proteins.

An article [36] presented whole protocol in the supplement, where the authors used maceration in a buffer consisting of 7 M urea + 2 M thiourea + 4% CHAPS + 65 mM DTT. This was followed by several centrifugation steps, ending with dialysis against ammonium bicarbonate. The resulting extract was then digested with trypsin. Maceration in the above buffer is particularly effective in preparing protein samples for proteomic analysis by ensuring that proteins are completely denatured, solubilized, and reduced. The buffer facilitates efficient extraction and processing of proteins from complex biological samples, enabling high‐quality proteomic data.

Proteomic analysis can be performed using two basic approaches: bottom‐up and top‐down. Tables 2 and 3 gives an overview of published papers for the analysis of amyloid proteins and M‐proteins from biological matrix. Bottom‐up approach was used in paper [6, 23, 29, 30, 31, 32, 33, 34, 35, 36, 37, 38, 40, 41, 42], and top‐down approach was used in only one paper [39]. Bottom‐up proteomics is the most commonly used method, especially when working with complex samples such as FFPE blocks [6, 30, 31, 32, 33, 34, 35], native tissues [37, 38], and adipose tissue [6, 29, 36]. This method involves the enzymatic digestion of proteins into smaller peptides, allowing detailed and sensitive analysis of highly complex protein mixtures. Its main advantages are higher sensitivity and the ability to analyze a wide range of proteins simultaneously. However, this approach can lose information about the overall structure of proteins and PTMs, which can be a limitation for studying certain aspects of proteins (Table 4).

**TABLE 3:** Review of selected publications for the determination of multiple myeloma M‐protein in different tissue types.

No.	Matrix	Identification Quantification	Instrumentation	Column	LOD LOQ	Analytes	Sample Preparation	Software	*n*
[40]	Serum	Identification Quantification	MALDI‐TOF‐MS Ig‐LC‐MS	n/a	n/a	M‐protein FLC	n/a	n/a	24
[41]	Serum	Quantification	Vanquish UHPLC coupled to a Q Exactive HF‐X hybrid quadrupole orbitrap mass spectrometer	n/a	LOD: n/a LOQ: 1.95–3.52 μg/mL	M‐protein	Sample preparation is described in the publication [35]	Xcalibur BioPharma Finder	19
[42]	Serum	Identification Quantification	MALDI‐TOF‐MS and LC‐MS	n/a	LOD: 15 μg/mL	M‐protein	n/a	n/a	585
[23]	Bone marrow and plasma	Identification Quantification	Thermo UltiMate 3000 nano system coupled in‐line with the Thermo Orbitrap Fusion Tribrid mass spectrometer (Plasma) MxP Quant 500 kit with SCIEX QTRAP 6500plus mass spectrometer (Medulla)	PepMap100 (C18, 300 μm × 5 mm) and Acclaim PepMap 100 (75 μm × 50 cm, 3 μm bead diameter) columns	n/a	Various MM proteins	Various preparations for medullary and blood samples using tryptic digestion (for more information see [36])	n/a	17

Abbreviation: n/a, not available.

**TABLE 4:** Pros and cons of commercially available mass analyzers in proteomic analysis [20, 86, 87, 88, 89, 90, 91, 92, 93].

Analyzer	Identification Quantification	Reasons for use in identification	Reasons for non‐use in quantification	Reasons for use in quantification	More information
Q‐TOF	Identification	High resolution, accurate mass measurements, fast scanning	Limited dynamic range, higher cost	Not typically used for quantification due to limited sensitivity	Used in proteomics for detailed protein characterization
Orbitrap	Identification	Extremely high resolution, accurate mass measurements	Slower scanning, higher cost	Not typically used for quantification due to slower scanning	Ideal for identifying complex mixtures and protein structure
MALDI‐TOF	Identification	Fast analysis, ability to analyze large biomolecules	Lower resolution than Q‐TOF and Orbitrap, matrix may interfere	Not suitable for quantification	Used for rapid analysis and identification of proteins and peptides
FT‐ICR	Identification	Highest resolution and accuracy available	Very high cost, difficult to maintain	Not suitable for quantification due to high cost and complexity	Used for detailed characterization and analysis of complex mixtures
QqQ	Quantification	Not the main choice for identification due to lower resolution	High sensitivity and specificity, ideal for quantification	High sensitivity, MRM capability, selective reaction monitoring	Used for accurate quantification of proteins and peptides
Linear Q trap	Identification Quantification	Ability to do MS^n^ analysis, high sensitivity	Lower resolution than Orbitrap and Q‐TOF	High sensitivity, combination with other analyzers	Suitable for quantification and identification steps
TOF	Identification Quantification	Wide mass range, fast scanning	Lower resolution than Orbitrap and Q‐TOF	Rapid screening analysis	Used for rapid quantification and screening analysis
IT (ion trap)	Identification Quantification	Ability to do MSn analysis, low cost	Lower resolution than Orbitrap and Q‐TOF	High sensitivity for fragmentation analysis	Suitable for combination with other analyzers and fragmentation analysis
Q‐Exactive (Quadrupole + Orbitrap)	Identification Quantification	High resolution, accurate mass measurements, MS/MS analysis capability	Higher costs and complexity of maintenance compared to QqQ	High sensitivity, precise quantification capability, suitable for complex samples	Used for detailed characterization and quantification of proteins, often in proteomics

On the other hand, top‐down proteomics focuses on the analysis of intact proteins, providing direct information about their primary structure, PTMs, and protein variants. Although it provides a more comprehensive view of protein structure, this method is technically more demanding, requires highly specialized equipment and is generally less effective when analyzing highly complex samples such as tissue lysates. In the literature, most authors working with FFPE blocks or adipose tissue use the bottom‐up approach because it is less time consuming and better suited for handling complex samples. Exceptions are studies such as Gonzalez Suarez et al. [37], which do not provide information on native tissue processing, and Lin et al. [39], which do not use classical proteomic approaches.

Overall, both methods have specific advantages and disadvantages depending on the type of sample and the desired information. Bottom‐up is more suitable for broad detection and quantification of proteins in complex mixtures, whereas top‐down is invaluable for detailed characterization of intact proteins and their modifications.

### 4.2 Proteomic Approaches in Multiple Myeloma

Proteomic approaches in multiple myeloma involve the comprehensive analysis of proteins expressed in this type of cancer, providing insights into disease mechanisms. There are not many articles focusing on the analysis of human blood for this purpose. In clinical practice, blood samples are routinely used to measure MRD in this disease; however, flow cytometry or next‐generation sequencing (NGS) are the main techniques used. NGS offers the highest sensitivity and specificity and is ideal for deep molecular analysis, but it is more expensive and technically and bioinformatically challenging. Flow cytometry is a very efficient, fast, and relatively inexpensive method with high sensitivity and specificity, but it may have limited ability to detect very low levels of MRD. Therefore, efforts are being made to incorporate MS into the routine measurement of this disease because it provides a good compromise between sensitivity and specificity and allows quantitative analysis but is technically more challenging and expensive than flow cytometry.

The authors of papers [40, 41, 42] used serum as a matrix for M‐protein analysis, and the authors of paper [23] used plasma, in addition, they also studied bone marrow. Using plasma as a protein‐rich matrix instead of serum allowed the authors to identify distinct protein and metabolite signatures of multiple myeloma. This distinction is crucial, because plasma can provide a more complete protein profile due to the presence of coagulation factors that are removed in serum. However, the use of plasma may introduce additional complexity in sample preparation and variability in results due to clotting factor interference. The authors made extensive use of the MALDI‐TOF combination [40, 42], which offers high‐throughput capabilities and simplicity in sample preparation, making it suitable for large‐scale studies. However, its resolution and ability to detect low abundant proteins in complex matrices is the main limiting factor that separates this technique from others. On the other hand, we can find other more sensitive tandem arrangements such as the combination of orbitrap [23, 41]. Orbitrap can provide higher mass accuracy and better resolution in this regard, resulting in more detailed analysis of complex matrices, but it requires more sophisticated instrumentation and may be less accessible. Conversely, the combination of quadrupole and linear ion trap used in [23] offers greater flexibility in detecting a wider range of molecular weights but may be more time consuming and require more complex sample preparation. Additionally, Ig‐LC‐MS technique used in article [40] is a method specialized for measuring immunoglobulin light chains, offered targeted insights into specific immunological biomarkers, although it is more focused and may not capture the broader proteomic profile observed with other MS techniques.

Some authors also reported limits of detection or quantification for their methods. The article [41] dealing with M‐protein analysis gives LOQs of 1.95–3.52 μg/mL for five out of six patients (patient 6 had a LOQ of 16.3 μg/mL, which the author explains by increased background noise). The author [42] reports an LOD for M‐protein identification of 15 μg/mL, which is a significant decrease in sensitivity compared to the results in article [41].

Tryptic digestion was used for sample preparation [23]; details of the preparation are given in Table 3. The bottom‐up approach was considered as a proteomic method mainly due to the use of digestion as described in article [23]. In article [41], the authors aimed at top‐down proteomics because of the analysis of intact proteins without a digestion step.

The analysis of multiple myeloma from blood can be challenging, especially because of the specific protein sequence in patient samples, so it is necessary to sequence each patient separately. For this reason, some papers do not analyze as many samples as the amyloid studies. The largest number of samples was analyzed in article [42] with 585 samples. MS screening showed a total of 66 positive samples.

Regarding the sample preparation, the authors have different approaches. For example, the authors [41] used preparation in a PCR plate where all incubation, washing, and reduction took place. This type of preparation was demanding in terms of the number of steps required to prepare the samples. Incubation and reduction of samples took 30 min each. Another paper [23] focused on a less demanding preparation in this respect, using plasma for immunodepletion with immunodepletion resin for 60 min, followed by centrifugation and protein digestion. The final step was acidification with 2% TFA in 20% ACN. In this article, the authors also focused on the use of both plasma and bone marrow samples for M‐protein determination. In the article, it is reported that in bone marrow, they found 225 proteins with a significant differential abundance between bone marrow mononuclear cells, whereas in plasma they found 22 proteins with a significant differential abundance.

## 5 Trends in Liquid Chromatography for the Analysis of Amyloidosis and Multiple Myeloma

Column separation in LC‐MS protein analysis is critical to achieving high accuracy and reliability of results. The choice of columns and sample preparation varies widely in proteomic studies, especially in the analysis of multiple myeloma and amyloidosis. When analyzing complex biological samples containing hundreds to thousands of different proteins, it is essential to effectively separate these proteins into individual fractions, with each peak corresponding to a specific protein or peptide. This separation allows their accurate identification and quantification by MS. In addition, good separation increases the sensitivity of the analysis by concentrating analytes into narrower peaks, making it easier to detect low concentrations of proteins. Conversely, poor separation can lead to overlapping signals in the MS detector, reducing the accuracy and sensitivity of detection and increasing the risk of interferences such as ion suppression. This in turn increases the risk of incorrect protein identification, especially in complex mixtures where similar peptides may elute together. Therefore, a high‐quality chromatographic separation not only ensures better resolution and sensitivity but also improves the reproducibility and accuracy of the analysis, which is essential for the validity of the results.

The trend in LC‐MS column chromatography of proteins, with a focus on amyloidosis and multiple myeloma in biological matrices, is to reduce column internal diameters, which requires lower mobile phase flow rates. This results in less sample dilution and consequently increased method sensitivity by concentrating analytes into narrower peaks. This trend is supported by the increasing use of micro‐ and nano‐LC techniques, which operate at flow rates in the micro‐ to nanoliters per minute, rather than the traditional milliliters per minute. Thanks to this technique, the methods benefit from both increased sensitivity and stable ionization, resulting in improved analyte transfer to the mass detector and thus improving overall data quality. These advantages have been well documented in published proteomic studies (Table 2) [6, 29, 31, 32, 33, 34, 36]. However, smaller columns and lower flow rates come with some trade‐offs. By reducing the internal diameters of the columns, there is a greater requirement for precise control and optimization of flow to avoid blockages and other associated problems. Shrinking columns provide a great solution in this regard if we do not achieve the required sensitivity; however, they can be further prone to problems associated with carryover, especially when dealing with such complex protein‐rich matrices.

Regarding columns for proteomic analysis, different types of columns can be used. One of the most commonly used types are reversed‐phase (RP) columns, which use hydrophobic interactions between the analyte and the stationary phase. This method has been extensively applied in amyloid and multiple myeloma research, with C18 columns featuring in most studies [31, 33, 34, 35, 36]. Due to hydrophobic interactions, RP columns can efficiently separate nonpolar proteins. However, a limitation may be the use of these columns in the separation of inversely hydrophilic analytes, which may subsequently lead to separation losses. This drawback is particularly shown in the paper [39], which attempts to exploit the use of HILIC columns. It is most likely that separation on C18 columns in particular has been utilized for analysis of MM, especially due to their abovementioned hydrophobic protein–stationary phase interactions [6, 29, 31, 32, 33, 34, 36]. .Another option that can be used are ion‐exchange columns used in multidimensional liquid chromatography, also known as the MudPIT technique. This technique combines the use of ion‐exchange and RP columns in several dimensions. A paper dealing with the analysis of amyloid from adipose tissue [36] used this technique and utilized a preparative SCX ion‐exchange column followed by a C18 separation column. However, the complexity of this technique may limit its wider application due to the increased time and resource requirements compared to simpler methods such as one‐dimensional RP separation. In articles focusing on amyloid typing, different types of trapping columns have been used, for example, Optipak trap filled with Magic C8 beads [35] and C18 Acclaim PepMap Nano Trap [35]. The use of these trapping columns prior to the main separation is crucial for enhancing the efficiency of peptide separation. The Optipak trap packed with Magic C8 beads utilizes moderate hydrophobic interactions, which can be particularly beneficial for capturing medium hydrophobicity peptides. However, the use of this type of precolumn can be tricky, especially with the loss of retention of highly hydrophobic or highly hydrophilic peptides, which can potentially affect resolution. On the other hand, the use of the C18 Acclaim PepMap Nano trapping column offers strong hydrophobic interactions due to the C18 stationary phase and thus makes it more suitable for trapping and concentrating highly hydrophobic peptides, but on the other hand, there is a risk of eluting highly hydrophilic peptides. Necessarily it should be mentioned that also in the analysis of multiple myeloma, there is the use of pre‐columns, namely, PepMap column [23] and PepMap precolumns, known for their robust performance in peptide retention under low flow conditions. However, as with all trapping columns, their efficiency can depend on the specific peptide properties, flow rates, and mobile phase conditions used in the analysis. Unfortunately, with regard to the further use of trapping columns in multiple myeloma analysis, the authors were not very forthcoming.

Regarding column size varied, with the most commonly used length being 15 cm [23, 33, 34, 35], but shorter columns were also found, up to 10 cm in an article dealing with MudPIT analysis from adipose tissue [36] or a 5‐cm HILIC column for analysis of beta‐amyloid from CSF fluid. Shorter columns can reduce analysis time but can also compromise resolution, especially in highly complex proteomic samples where overlapping peaks can make detection impossible. Regarding the internal diameters, the use of NanoFlow‐LC methods has also resulted in a dramatic reduction of internal diameters to 75 μm [33, 34, 35]. This reduction in diameter offers several advantages, particularly in terms of sensitivity. Smaller internal diameters concentrate the analyte in a narrower flow path, resulting into sharper peaks and higher sensitivity, which is critical for detecting low‐abundance proteins in complex biological samples.

Particle sizes is another critical parameter, in many studies sizes range from 3 to 5 μm [23, 34, 36, 39], which is in contrast to classical UHPLC applications using 1.7‐μm particles. The larger particle sizes in nano‐LC methods are necessary to accommodate the low flow rates and smaller column diameters, though they may reduce separation efficiency compared to the smaller particles in UHPLC.

The mobile phase is performed under gradient elution for most articles [23, 31, 32, 33, 35, 36, 39]. If the authors stated so, they used mobile phases with water and acetonitrile with additions of formic acid, which supports effective peptide elution and ionization [23, 31, 32, 33, 35, 36, 39].

However, the lack of detailed information about mobile phase conditions in some studies [6, 29, 30, 34, 37, 38, 40, 41, 42] leaves room for potential inconsistencies in comparative analyses.

## 6 MS

In recent decades, MS‐based proteomics has become a key tool for the detailed investigation of molecular mechanisms in several diseases, including amyloidosis [43], multiple myeloma [44], Alzheimer's disease [45, 46], Parkinson's disease [47], cardiovascular diseases [48, 49], various types of cancer [50, 51, 52], diabetes [53], autoimmune diseases such as rheumatoid arthritis [54], and neurodegenerative diseases such as Huntington's disease [55, 56]. In addition, MS‐based proteomics has been instrumental in the study of infectious diseases, including HIV/AIDS [57] and hepatitis [58, 59], and kidney diseases such as glomerulonephritis [60]. Due to its high sensitivity, resolution, and mass accuracy, MS allows the identification and quantification of thousands of proteins in complex biological samples. The combination of liquid chromatography and tandem mass spectrometry (LC‐MS/MS) has become the gold standard for complex proteomic analysis, allowing efficient separation of protein mixtures prior to detection and identification—critical when working with clinical samples.

Significant advances in MS instrument configurations—particularly the development of modern high‐ and ultra‐high‐resolution mass spectrometers (HRAM) such as time‐of‐flight (TOF) MS, Fourier transform cyclotron resonance (FT‐ICR) MS, and Orbitrap—have taken proteomics to new heights and facilitated the transition of MS technology from analytical laboratories to clinical practice. Hybrid MS systems such as the Q‐Exactive (combining quadrupole and Orbitrap) [6, 23, 31, 32, 34, 41] and the LTQ‐Orbitrap (integrating linear ion trap, quadrupole, and Orbitrap) [6, 23, 33, 35, 36, 39] are widely used in both research and clinical laboratories. These instruments achieve exceptional performance through advanced modifications in various components, such as ion sources, ion transfer optics, and instrument tuning, which collectively increase sensitivity and detection speed. Optimized signal processing and electronics further enhance their capabilities, enabling both qualitative and quantitative analysis with high accuracy and resolution. These high‐resolution capabilities are critical in proteomics for identifying and quantifying low‐abundance proteins, detecting PTMs, and distinguishing between proteins with very similar masses. In addition, advanced ion optics and improved scan speeds enable faster data acquisition, which is essential when analyzing large numbers of samples or conducting high‐throughput studies in clinical settings.

Proteomic analysis primarily employs two complementary strategies. The first aims to comprehensively identify proteins in biological samples, providing a broad view of the proteome. Recent advances in MS and bioinformatics have greatly expanded the scope of this approach, enabling the identification of thousands of proteins in complex biological matrices such as plasma, urine, CSF, and tissue extracts. For example, state‐of‐the‐art MS techniques have facilitated the identification of more than 10 000 proteins in human plasma [61] and more than 3000 proteins in urine [62]. A Q‐Exactive mass spectrometer was used to analyze the proteomic profile of serum from patients with refractory multiple myeloma, and 632 proteins were identified, 52 of which showed significant differences between different patient groups [63]. Dasari et al. [6] reviewed data from 16 175 samples analyzed by MS to subtype 21 amyloid proteins, demonstrating 99% specificity in amyloidosis typing and identifying rare mutations associated with hereditary forms. Chanukuppa et al. [24] used Sequential Window Acquisition of All Theoretical Mass Spectra (SWATH‐MS) and complementary proteomic techniques to identify 279 differentially expressed proteins in bone marrow interstitial fluid and 116 in serum from multiple myeloma patients, revealing potential biomarkers for disease diagnosis and pathophysiology. This approach offers advantages such as high reproducibility, comprehensive proteome coverage, and the ability to analyze complex biological fluids, overcoming the specificity limitations of targeted MS methods and enabling broader biomarker discovery compared to studies focused solely on purified plasma cells. Brambilla et al. [36] demonstrated the application of multidimensional protein identification technology (MudPIT) for proteomic typing of systemic amyloidoses using subcutaneous fat aspirates from 26 patient samples. By combining MS/MS with 2D chromatography, the study successfully identified amyloidogenic proteins—light chains κ (LC‐κ), λ (LC‐λ), TTR, and SAA—as well as associated fibrillogenesis‐related proteins, including clusterin and apolipoprotein E. The MudPIT‐MS analysis compared patient samples with controls to develop a diagnostic algorithm based on normalized abundance ratios (α‐values) for subtype classification. Validation against immunoelectron microscopy confirmed complete agreement in amyloid type assignment. The workflow eliminates the need for tissue fractionation, reduces noise from plasma proteins, and enables automated high‐throughput analysis of complex protein samples. Dunphy et al. [23] used label‐free LC‐MS/MS and targeted metabolomics to analyze bone marrow mononuclear cells (BMNCs) and plasma from multiple myeloma (MM) patients with and without extramedullary spread (EMM). Proteomic analysis identified 225 differentially abundant proteins in BMNCs and 22 in plasma, whereas metabolomics revealed 31 altered metabolites, primarily lipids, in EMM plasma. Key biomarkers, including VCAM1, HGFA, and PEDF, were validated as plasma markers to discriminate EMM from MM, achieving an AUC of 1.0 in ROC analysis. Proteomic pathways highlighted mechanisms such as integrin‐mediated signaling and Rap1 signaling that drive EMM progression. Analyses were performed using Thermo Orbitrap Fusion Tribrid MS for BMNCs, Q‐Exactive MS for plasma, and SCIEX QTRAP 6500plus for metabolomics. This integrative approach provides insight into the molecular basis of EMM and suggests clinically relevant biomarkers for diagnosis. Holub et al. [35] compared IHC with laser microdissection–liquid chromatography–tandem mass spectrometry (LMD‐LC‐MS/MS) for amyloid typing in 22 FFPE tissue samples from 11 patients with systemic amyloidosis. LMD‐LC‐MS/MS accurately identified amyloid subtypes in all samples, outperforming IHC, which was accurate in only 36% of cases and prone to false positives, particularly for TTR and SAA. LMD‐LC‐MS/MS demonstrated superior sensitivity, specificity and reproducibility across tissue types and reliably identified amyloidogenic and associated proteins. Whereas IHC remains cost‐effective and widely used, LMD‐LC‐MS/MS provides a more accurate and comprehensive approach, particularly in cases where IHC results are inconclusive. This study highlights the potential of proteomics to improve the diagnosis of amyloidosis.

The second strategy focuses on the targeted identification and quantification of specific proteins that often serve as biomarkers of disease. This targeted approach is critical for clinical applications, as precise measurement of disease‐associated proteins aids in diagnosis, prognosis, and monitoring of treatment response. In the field of amyloidosis, more than 130 potentially clinically relevant amyloidogenic proteins have been identified [64]. However, routine clinical practice typically focuses on a select few, including SAA, TTR, immunoglobulin light chains (κ and λ), and β2‐microglobulin, due to their established roles in disease pathology and management [8, 65]. Similarly, in multiple myeloma, the detection and quantification of monoclonal (M) proteins, including immunoglobulin heavy and light chains, is essential for diagnostic and monitoring purposes [66, 67]. Nevone et al. [68] investigated N‐glycosylation in κ‐type immunoglobulin light chains in AL amyloidosis. The study used the Q‐Exactive spectrometer to identify and analyze specific glycosylated light chain variants that tend to form amyloid fibrils. Specific N‐glycosylation patterns within the FR3 region were identified that contribute to the risk of amyloidosis progression in multiple myeloma patients. Two landmark studies have advanced the development and clinical application of EXENT‐MS, a MALDI‐TOF‐MS‐based technology for the detection and quantification of monoclonal proteins (M‐proteins), highlighting its transformative potential in the noninvasive monitoring of multiple myeloma. In the first study, Kubicki et al. [42] introduced EXENT‐MS as a noninvasive alternative to bone marrow biopsy during maintenance therapy for multiple myeloma. The method proved valuable in assessing measurable residual disease (MRD) and predicting progression‐free survival. With a detection limit of 0.015 g/L, EXENT‐MS accurately quantifies M‐proteins across immunoglobulin isotypes (IgG, IgA, and IgM) and offers rapid processing and ease of implementation compared to more complex methods such as LC‐MS/MS. The integration of automated sample processing ensures standardized and efficient workflows, making it ideal for routine clinical practice. However, its accuracy depends on patient‐specific calibration, as the unique mass spectra of M‐proteins vary according to immunoglobulin isotype and structural mutations. Calibration requires previous patient samples to establish a reference spectrum, which is critical for reliable longitudinal tracking of M‐proteins. Despite this limitation, EXENT‐MS represents a significant innovation in noninvasive diagnostics with the potential to significantly improve clinical care. In the second study, Barnidge et al. [69] compared EXENT‐MS with LC‐MS/MS (Triple‐TOF) for the detection and quantification of M‐proteins in serum samples from patients with suspected or diagnosed multiple myeloma. In addition to M‐proteins, this study analyzed PTMs such as glycosylation to assess protein heterogeneity. Sensitivity was a major focus: Whereas EXENT‐MS achieved a detection limit of 0.015 g/L, sufficient for most clinical applications, LC‐MS/MS demonstrated significantly greater sensitivity, identifying trace protein concentrations below 0.001 g/L. This capability enabled LC‐MS/MS to detect M‐proteins in samples where EXENT‐MS did not, illustrating the complementary strengths of the two technologies. EXENT‐MS is characterized by high throughput and streamlined automation, whereas LC‐MS/MS offers unparalleled sensitivity and detailed molecular characterization. This technology has also been featured in other publications [70, 71, 72], highlighting its growing role in clinical and research settings. Multi‐method LC‐MS/MS approaches for the quantification of pathological proteins offer significant advantages over single‐method analyses, particularly for monitoring a wide range of diseases. Recent advances by several authors have demonstrated multiplexed methods capable of analyzing tens to hundreds of proteins from complex biological matrices, thereby increasing diagnostic accuracy and throughput [73, 74, 75, 76, 77]. Kuzyk et al. [78] with a method to quantify 45 plasma proteins associated with cardiovascular, cancer, and inflammatory diseases. Using MRM‐based LC‐MS/MS with stable isotope‐labeled peptides, the study achieved attomole‐level LOQs for 27 proteins and CVs below 10% for 44 assays. Targeted MS techniques, such as multiple reaction monitoring (MRM) and parallel reaction monitoring (PRM), have been instrumental in the quantification of low‐abundance proteins in complex biological matrices [78, 79]. These techniques have been successfully applied to quantify amyloidogenic proteins and M‐proteins in clinical samples, significantly improving the accuracy of amyloidosis and multiple myeloma diagnosis [80].

Data acquisition strategies are critical in mass spectrometry‐based proteomics, with data‐dependent acquisition (DDA) and data‐independent acquisition (DIA) representing two dominant approaches [81, 82, 83, 84]. Each offers unique strengths for protein analysis, enabling advances in diagnostics and biological research. DDA is based on selecting the most abundant precursor ions in an initial MS1 scan for subsequent fragmentation and analysis in MS2. This “top‐n” selection ensures high quality spectra for targeted precursors, but is inherently stochastic. As a result, DDA can introduce missing data when precursor ion intensities vary between samples. Its main advantage is that it produces clean MS2 spectra that are well suited for identifying PTMs and performing open or targeted searches. However, its stochastic nature limits its reproducibility, especially in highly complex samples [85]. Kelstrup et al. [86] optimized DDA for shotgun proteomics and identified over 4000 proteins in a 3‐h LC‐MS/MS analysis using a quadrupole Orbitrap mass spectrometer, demonstrating the method's ability to identify proteins at high resolution. Other publications have explored DDA proteomic analysis of proteins in biological matrices [87, 88, 89, 90, 91]. DIA overcomes these limitations by fragmenting all precursor ions within defined m/z windows, providing comprehensive and unbiased data collection. DIA ensures consistent sampling of peptides across samples, significantly reducing missing values and improving reproducibility, especially in large cohort studies. The increased complexity of DIA spectra requires advanced computational tools and spectral libraries for data interpretation. Innovations such as pseudo‐MS2 spectra generation and machine learning–enhanced analysis have mitigated these challenges, enabling reliable quantification of thousands of proteins. Other studies have used DIA to analyze clinically relevant proteins [92, 93].

IMS has revolutionized MS workflows, particularly DDA and DIA methods. By separating ions based on their CCS, IMS introduces an additional dimension of separation that enhances molecular resolution and specificity. This unique capability allows differentiation of isobaric and isomeric ions, addressing critical challenges in proteomic analysis such as spectral overlap and co‐elution. IMS significantly improves both protein identification and quantification by simplifying mass spectra. This simplification leads to better coverage of identified proteins, reproducibility, and sensitivity of analyses. In addition, IMS enables researchers to delve deeper into the structural intricacies of peptides and proteins, including the characterization of PTMs. The integration of IMS into MS workflows has led to significant advances in proteomic research. For example, Jiang et al. [94] demonstrated that IMS doubled the number of quantifiable proteins in plasma samples, identifying more than 1000 proteins compared to approximately 500 using conventional LC‐MS/MS. Similarly, McMillen et al. [95] and Aftab et al. [96] reported improved protein detection and resolution in tryptic peptide and tissue proteomics, respectively. In the field of clinical proteomics, IMS has proven invaluable. Dunphy et al. [23] used IMS to separate amyloid isoforms, providing insight into structural variations associated with disease progression in multiple myeloma and amyloidosis. Umapathy et al. [97] integrated IMS with DIA for salivary proteomics, identifying novel biomarkers such as matrix metalloproteinases for oral cancer. Large‐scale studies using trapped ion mobility spectrometry (TIMS) have greatly improved the detection and quantification of plasma proteins, facilitating biomarker discovery and advancing both basic and clinical research. In addition, Tomioka et al. [98] compared digestion workflows with and without IMS and found significant improvements in the detection and quantification of host cell proteins in bottom‐up proteomics.

Automated systems for proteomic analysis have significantly advanced clinical diagnostics and research by improving efficiency, sensitivity, and reproducibility. The study by Dasari et al. [99] examines the MASS‐FIX system, an advanced automated MALDI‐TOF‐MS method for the detection and isotyping of monoclonal proteins (M‐proteins) associated with plasma cell disorders such as multiple myeloma and amyloidosis. The automation focuses on the sample preparation phase, which is performed by robotic liquid handlers. This includes immunoenrichment of immunoglobulin subclasses and light chains, followed by automated spotting onto MALDI plates. MALDI‐TOF‐MS analysis and data interpretation are also automated, supported by software capable of efficiently identifying abnormal mass patterns indicative of M‐proteins. MASS‐FIX was shown to be superior to traditional immunofixation electrophoresis (IFE), offering higher sensitivity and fewer false negatives, particularly in the detection of glycosylated light chains, which are associated with a higher risk of light chain amyloidosis (AL) and other plasma cell disorders. It also demonstrated efficiency in the detection of therapeutic monoclonal antibodies, reducing potential diagnostic interference. With a sample recall rate of less than 1.5% and a 30% increase in throughput over IFE, MASS‐FIX exemplifies the integration of automated MS into clinical workflows to improve diagnostic accuracy and laboratory efficiency. Other innovations include the autoPOTS platform developed by Liang et al. [100], which automates low‐input proteome profiling with high precision, making it suitable for streamlined workflows in clinical and research laboratories. Similarly, SP3, presented by Müller et al. [101], automates sample preparation to improve reproducibility and scalability in proteomic workflows, especially for low‐input samples. Messner et al. [102] demonstrated the potential of ultra‐high‐throughput proteomics with automated systems for the analysis of COVID‐19 patient plasma, demonstrating the speed and scalability required for large‐scale diagnostic studies. In addition, high‐throughput LC‐MS/MS platforms, as exemplified by Smit et al. [103], have been implemented to provide accurate and efficient quantitative proteomic analyses that meet the needs of modern clinical laboratories. Together, these automated systems highlight the transformative role of automation in proteomic workflows, providing scalable, robust, and clinically relevant solutions for disease diagnostics and biomarker discovery.

## 7 Database

After acquiring fragmentation spectra from MS, the first critical step is the identification of peptide sequences. This can be accomplished by two main strategies: searching protein sequence databases [104, 105, 106] or de novo peptide sequencing [107, 108, 109]. Although MS generates complex spectral data, it does not inherently provide direct information about protein identity or quantity [110, 111]. Specialized software tools and bioinformatics methods are essential to interpret these spectra and transform raw data into actionable insights suitable for further analysis [112]. Protein databases, such as UniProt (Universal Protein Resource) [113], SwissProt, and NCBI, play a central role in this process. UniProt is a carefully curated database containing comprehensive protein sequence data, including isoforms, PTMs, and functional annotations. By comparing experimental MS/MS spectra with theoretical spectra generated from these databases, software tools enable accurate protein identification, which is essential for subsequent interpretation.

A variety of software programs facilitate this analysis, each optimized for specific tasks. Tools such as Mascot [104] use probabilistic algorithms for peptide identification and take PTMs into account, making them highly reliable. SEQUEST [105], one of the first MS/MS algorithms, is still widely used, especially when integrated with Thermo Fisher Scientific systems. Open‐source software such as X!Tandem [106] offers flexibility in search parameters, making it suitable for large data sets. To increase accuracy, researchers often use multiple programs in parallel; for example, the combination of Mascot, SEQUEST, and X!Tandem has been used to compensate for individual limitations and achieve more comprehensive results [114, 115, 116, 117, 118]. Less common tools such as Crux [109] are valued for their speed and efficiency [119], whereas proprietary solutions such as Thermo Fisher Scientific's Xcalibur, SCIEX's ProteinPilot, and Waters' ProteinLynx Global Server (PLGS) are tailored to specific instrumentation [120].

For protein quantification, whether relative or absolute, tools such as MaxQuant [121], which integrates the Andromeda algorithm for high‐accuracy quantification using both label‐free and labeled approaches, are available. Thermo Fisher Scientific's Proteome Discoverer combines tools for identification and quantification, supporting different experimental workflows and increasing the versatility of proteomic analyses [112].

In diseases such as amyloidosis and multiple myeloma, accurate protein identification and quantification are essential for understanding disease mechanisms and improving diagnostics. For example, immunoglobulin light chains, a hallmark of multiple myeloma, can be identified and quantified using specialized software and reference sequences in databases such as UniProt [122]. Similarly, in amyloidosis, MS combined with specialized software and the UniProt database allows the identification of proteins that form amyloid deposits, providing insight into disease pathogenesis and potential therapeutic targets [122]. Despite significant advances, challenges remain in integrating MS data with databases, including incomplete protein annotations and inconsistencies in spectral libraries. Future developments should focus on expanding databases with high‐resolution data and fostering collaboration between computational and experimental scientists. Such efforts are critical to improving disease diagnosis and biomarker discovery. Databases and computational tools are integral to proteomic workflows, transforming MS data into practical and actionable information. Their synergy with advanced MS instrumentation accelerates biomarker discovery and improves our understanding of diseases such as amyloidosis and multiple myeloma.

## Data Availability

Data sharing not applicable to this article as no datasets were generated or analyzed during the current study.

## References

[1] A.‐H. Emwas , M. Alghrably , M. Dhahri , et al., “Living With the Enemy: From Protein‐Misfolding Pathologies We Know, to Those We Want to Know,” Ageing Research Reviews 70 (2021): 101391, 10.1016/j.arr.2021.101391. 34119687

[2] G. A. Khoury , R. C. Baliban , and C. A. Floudas , “Proteome‐Wide Post‐translational Modification Statistics: Frequency Analysis and Curation of the Swiss‐Prot Database,” Scientific Reports 1 (2011): 90, 10.1038/srep00090. 22034591 PMC3201773

[3] S. Ramazi and J. Zahiri , “Post‐translational Modifications in Proteins: Resources, Tools and Prediction Methods,” Database 2021 (2021): baab012, 10.1093/database/baab012. 33826699 PMC8040245

[4] L. Guan , W. Su , J. Zhong , and L. Qiu , “M‐Protein Detection by Mass Spectrometry for Minimal Residual Disease in Multiple Myeloma,” Clinica Chimica Acta 552 (2024): 117623, 10.1016/j.cca.2023.117623. 37924928

[5] J. A. Gilbertson , J. D. Theis , J. A. Vrana , et al., “A Comparison of Immunohistochemistry and Mass Spectrometry for Determining the Amyloid Fibril Protein From Formalin‐Fixed Biopsy Tissue,” Journal of Clinical Pathology 68 (2015): 314–317, 10.1136/jclinpath-2014-202722. 25637636

[6] S. Dasari , J. D. Theis , J. A. Vrana , et al., “Amyloid Typing by Mass Spectrometry in Clinical Practice: a Comprehensive Review of 16,175 Samples,” Mayo Clinic Proceedings 95 (2020): 1852–1864, 10.1016/j.mayocp.2020.06.029. 32861330

[7] S. Kumar , A. Dispenzieri , J. A. Katzmann , et al., “Serum Immunoglobulin Free Light‐Chain Measurement in Primary Amyloidosis: Prognostic Value and Correlations With Clinical Features,” Blood 116 (2010): 5126–5129, 10.1182/blood-2010-06-290668. 20798235 PMC3012533

[8] M. A. Gertz , “Immunoglobulin Light Chain Amyloidosis: 2018 Update on Diagnosis, Prognosis, and Treatment,” American Journal of Hematology 93 (2018): 1169–1180, 10.1002/ajh.25149. 30040145

[9] A. Macklin , S. Khan , and T. Kislinger , “Recent Advances in Mass Spectrometry Based Clinical Proteomics: Applications to Cancer Research,” Clinical Proteomics 17 (2020): 17, 10.1186/s12014-020-09283-w. 32489335 PMC7247207

[10] Z. Noor , S. B. Ahn , M. S. Baker , S. Ranganathan , and A. Mohamedali , “Mass Spectrometry–Based Protein Identification in Proteomics—A Review,” Briefings in Bioinformatics 22 (2021): 1620–1638, 10.1093/bib/bbz163. 32047889

[11] L. Bertamini , M. D'Agostino , and F. Gay , “MRD Assessment in Multiple Myeloma: Progress and Challenges,” Current Hematologic Malignancy Reports 16 (2021): 162–171, 10.1007/s11899-021-00633-5. 33950462

[12] S. Rozanova , K. Barkovits , M. Nikolov , C. Schmidt , H. Urlaub , and K. Marcus , “Quantitative Mass Spectrometry‐Based Proteomics: An Overview,” in Quantitative Methods in Proteomics, vol. 2228 (New York, NY: Springer US, 2021): 85–116. 10.1007/978-1-0716-1024-4_8 33950486

[13] D. Wenk , C. Zuo , T. Kislinger , and L. Sepiashvili , “Recent Developments in Mass‐Spectrometry‐Based Targeted Proteomics of Clinical Cancer Biomarkers,” Clinical Proteomics 21 (2024): 6, 10.1186/s12014-024-09452-1. 38287260 PMC10826105

[14] B. J. Smith , D. Martins‐de‐Souza , and M. Fioramonte , “A Guide to Mass Spectrometry‐Based Quantitative Proteomics,” in Pre‐Clinical Models, vol. 1916 (New York, New York, NY: Springer, 2019): 3–39. 10.1007/978-1-4939-8994-2_1 30535679

[15] A. Sinha and M. Mann , “A Beginner's Guide to Mass Spectrometry–Based Proteomics,” Biochemistry 42 (2020): 64–69, 10.1042/BIO20200057.

[16] W. J. Griffiths and Y. Wang , “Mass Spectrometry: From Proteomics to Metabolomics and Lipidomics,” Chemical Society Reviews 38 (2009): 1882–1896, 10.1039/b618553n. 19551169

[17] J. Cox and M. Mann , “Is Proteomics the New Genomics?,” Cell 130 (2007): 395–398, 10.1016/j.cell.2007.07.032. 17693247

[18] B. Aslam , M. Basit , M. A. Nisar , M. Khurshid , and M. H. Rasool , “Proteomics: Technologies and Their Applications,” Journal of Chromatographic Science 55 (2017): 182–196, 10.1093/chromsci/bmw167. 28087761

[19] S.‐E. Ong and M. Mann , “Mass Spectrometry–Based Proteomics Turns Quantitative,” Nature Chemical Biology 1 (2005): 252–262, 10.1038/nchembio736. 16408053

[20] M. Bantscheff , S. Lemeer , M. M. Savitski , and B. Kuster , “Quantitative Mass Spectrometry in Proteomics: Critical Review Update From 2007 to the Present,” Analytical and Bioanalytical Chemistry 404 (2012): 939–965, 10.1007/s00216-012-6203-4. 22772140

[21] D. R. Barnidge , S. Dasari , C. M. Botz , et al., “Using Mass Spectrometry to Monitor Monoclonal Immunoglobulins in Patients With a Monoclonal Gammopathy,” Journal of Proteome Research 13 (2014): 1419–1427, 10.1021/pr400985k. 24467232

[22] K. Thoren , S. Menad , G. Nouadje , and S. Macé , “Isatuximab‐Specific Immunofixation Electrophoresis Assay to Remove Interference in Serum M‐Protein Measurement in Patients With Multiple Myeloma,” Journal of Applied Laboratory Medicine 9 (2024): 661–671, 10.1093/jalm/jfae028. 38573925

[23] K. Dunphy , D. Bazou , M. Henry , et al., “Proteomic and Metabolomic Analysis of Bone Marrow and Plasma From Patients With Extramedullary Multiple Myeloma Identifies Distinct Protein and Metabolite Signatures,” Cancers 15 (2023): 3764, 10.3390/cancers15153764. 37568580 PMC10417544

[24] V. Chanukuppa , R. Taware , K. Taunk , et al., “Proteomic Alterations in Multiple Myeloma: A Comprehensive Study Using Bone Marrow Interstitial Fluid and Serum Samples,” Frontiers in Oncology 10 (2021): 566804, 10.3389/fonc.2020.566804. 33585190 PMC7879980

[25] E. M. O'Sullivan , et al., “Proteomic Identification of Saliva Proteins as Noninvasive Diagnostic Biomarkers,” in Difference Gel Electrophoresis, vol. 2596 (New York, NY: Springer US, 2023): 147–167. 10.1007/978-1-0716-2831-7_12 36378438

[26] B. Wisniowski and A. Wechalekar , “Confirming the Diagnosis of Amyloidosis,” Acta Haematologica 143 (2020): 312–321, 10.1159/000508022. 32544917

[27] S. De Meyer , J. M. Schaeverbeke , I. M. W. Verberk , et al., “Comparison of ELISA‐ and SIMOA‐Based Quantification of Plasma Aβ Ratios for Early Detection of Cerebral Amyloidosis,” Alzheimer's Research & Therapy 12 (2020): 162, 10.1186/s13195-020-00728-w. PMC7719262 33278904

[28] E. Muchtar , A. Dispenzieri , M. A. Gertz , et al., “Treatment of AL Amyloidosis: Mayo Stratification of Myeloma and Risk‐Adapted Therapy (mSMART) Consensus Statement 2020 Update,” Mayo Clinic Proceedings 96 (2021): 1546–1577, 10.1016/j.mayocp.2021.03.012. 34088417

[29] J. A. Vrana , J. D. Theis , S. Dasari , et al., “Clinical Diagnosis and Typing of Systemic Amyloidosis in Subcutaneous fat Aspirates by Mass Spectrometry‐Based Proteomics,” Haematologica 99 (2014): 1239–1247, 10.3324/haematol.2013.102764. 24747948 PMC4077087

[30] J. A. Vrana , J. D. Gamez , B. J. Madden , J. D. Theis , H. R. Bergen, III , and A. Dogan , “Classification of Amyloidosis by Laser Microdissection and Mass Spectrometry–Based Proteomic Analysis in Clinical Biopsy Specimens,” Blood 114 (2009): 4957–4959, 10.1182/blood-2009-07-230722. 19797517

[31] N. Abildgaard , A. M. Rojek , H. E. H. Møller , et al., “Immunoelectron Microscopy and Mass Spectrometry for Classification of Amyloid Deposits,” Amyloid 27 (2020): 59–66, 10.1080/13506129.2019.1688289. 31752543

[32] W. S. Phipps , K. D. Smith , H. Y. Yang , et al., “Tandem Mass Spectrometry–Based Amyloid Typing Using Manual Microdissection and Open‐Source Data Processing,” American Journal of Clinical Pathology 157 (2022): 748–757, 10.1093/ajcp/aqab185. 35512256 PMC9071319

[33] J. D. Theis , S. Dasari , J. A. Vrana , P. J. Kurtin , and A. Dogan , “Shotgun‐Proteomics‐Based Clinical Testing for Diagnosis and Classification of Amyloidosis,” Journal of Mass Spectrometry 48 (2013): 1067–1077, 10.1002/jms.3264. 24130009

[34] T. Rezk , J. A. Gilbertson , P. P. Mangione , et al., “The Complementary Role of Histology and Proteomics for Diagnosis and Typing of Systemic Amyloidosis,” Journal of Pathology: Clinical Research 5 (2019): 145–153, 10.1002/cjp2.126. 30740936 PMC6648380

[35] D. Holub , P. Flodrova , T. Pika , P. Flodr , M. Hajduch , and P. Dzubak , “Mass Spectrometry Amyloid Typing Is Reproducible Across Multiple Organ Sites,” BioMed Research International 2019 (2019): 1–9, 10.1155/2019/3689091. PMC6374819 30834260

[36] F. Brambilla , F. Lavatelli , D. di Silvestre , et al., “Reliable Typing of Systemic Amyloidoses Through Proteomic Analysis of Subcutaneous Adipose Tissue,” Blood 119 (2012): 1844–1847, 10.1182/blood-2011-07-365510. 21917755

[37] M. L. Gonzalez Suarez , P. Zhang , S. H. Nasr , et al., “The Sensitivity and Specificity of the Routine Kidney Biopsy Immunofluorescence Panel Are Inferior to Diagnosing Renal Immunoglobulin‐Derived Amyloidosis by Mass Spectrometry,” Kidney International 96 (2019): 1005–1009, 10.1016/j.kint.2019.05.027. 31447055

[38] S. Sethi , J. A. Vrana , J. D. Theis , et al., “Laser Microdissection and Mass Spectrometry–Based Proteomics Aids the Diagnosis and Typing of Renal Amyloidosis,” Kidney International 82 (2012): 226–234, 10.1038/ki.2012.108. 22495291 PMC3388518

[39] P. Lin , W. L. Chen , F. Yuan , et al., “An UHPLC–MS/MS Method for Simultaneous Quantification of Human Amyloid beta Peptides Aβ1‐38, Aβ1‐40 and Aβ1‐42 in Cerebrospinal Fluid Using Micro‐Elution Solid Phase Extraction,” Journal of Chromatography B 1070 (2017): 82–91, 10.1016/j.jchromb.2017.10.047. 29102244

[40] D. Foureau , M. Bhutani , F. Guo , et al., “Comparison of Mass Spectrometry and Flow Cytometry in Measuring Minimal Residual Disease in Multiple Myeloma,” Cancer Medicine 10 (2021): 6933–6936, 10.1002/cam4.4254. 34494717 PMC8525140

[41] S. Muccio , C. Hirtz , S. Descloux , et al., “A Sensitive High‐Resolution Mass Spectrometry Method for Quantifying Intact M‐Protein Light Chains in Patients With Multiple Myeloma,” Clinica Chimica Acta 552 (2024): 117634, 10.1016/j.cca.2023.117634. 37980975

[42] T. Kubicki , D. Dytfeld , D. Barnidge , et al., “Mass Spectrometry‐Based Assessment of M‐Protein in Peripheral Blood During Maintenance Therapy in Multiple Myeloma,” Blood Journal 144 (2024): 955–963, 10.1182/blood.2024024041. PMC11406170 38713888

[43] M. Spodzieja , S. Rodziewicz‐Motowidło , and A. Szymanska , “Hyphenated Mass Spectrometry Techniques in the Diagnosis of Amyloidosis,” Current Medicinal Chemistry 26 (2019): 104–120, 10.2174/0929867324666171003113019. 28971756

[44] N. Gupta , H. Kaur , and S. Wajid , “Renal Amyloidosis: An Update on Diagnosis and Pathogenesis,” Protoplasma 257 (2020): 1259–1276, 10.1007/s00709-020-01513-0. 32447467

[45] M. Chen , Y. Li , H. Lv , P. Yin , L. Zhang , and P. Tang , “Quantitative Proteomics and Reverse Engineer Analysis Identified Plasma Exosome Derived Protein Markers Related to Osteoporosis,” Journal of Proteomics 228 (2020): 103940, 10.1016/j.jprot.2020.103940. 32805449

[46] K. Hobin , L. Abou‐Zeid , I. B. Mendizabal , et al., “Investigation of the Concentration and Isotopic Composition of Cu, Fe and Zn in Human Biofluids in the Context of Alzheimer's Disease via Tandem and Multi‐collector Inductively Coupled Plasma‐Mass Spectrometry,” Journal of Trace Elements in Medicine and Biology 86 (2024): 127515, 10.1016/j.jtemb.2024.127515. 39241488

[47] O. Karayel , S. Virreira Winter , S. Padmanabhan , et al., “Proteome Profiling of Cerebrospinal Fluid Reveals Biomarker Candidates for Parkinson's Disease,” Cell Reports Medicine 3 (2022): 100661, 10.1016/j.xcrm.2022.100661. 35732154 PMC9245058

[48] X. Wang , W. Zhou , Z. Gao , and X. Lv , “Mass Spectrometry Analysis of S‐Nitrosylation of Proteins and Its Role in Cancer, Cardiovascular and Neurodegenerative Diseases,” TrAC Trends in Analytical Chemistry 152 (2022): 116625, 10.1016/j.trac.2022.116625.

[49] Y. Tang , S. Wang , W. Zhang , et al., “A Single‐Run, Rapid Polarity Switching Method for Simultaneous Quantification of Cardiovascular Disease‐Related Metabolites Using Liquid Chromatography–Tandem Mass Spectrometry,” International Journal of Mass Spectrometry 461 (2021): 116500, 10.1016/j.ijms.2020.116500.

[50] X. Cheng , W. Yu , Y. Liu , S. Jia , D. Wang , and L. Hu , “Proteomic Characterization of Urinary Exosomes With Pancreatic Cancer by Phosphatidylserine Imprinted Polymer Enrichment and Mass Spectrometry Analysis,” Journal of Proteome Research 24 (2024): 111–120, 10.1021/acs.jproteome.4c00508. 39392357

[51] Y. Li , et al., “Unlocking the Future of Colorectal cancer Detection: Advances in Screening Glycosylation‐Based Biomarkers on Biological Mass Spectrometry Technology,” Journal of Chromatography. A 1738 (2024): 465501, 10.1016/j.chroma.2024.465501. 39504704

[52] O. S. Yildirim , P. Yildiz , A. Karaer , J. Calleja‐Agius , and S. Ozcan , “Exploring the Protein Signature of Endometrial Cancer: A Comprehensive Review Through Diverse Samples and Mass Spectrometry‐Based Proteomics,” European Journal of Surgical Oncology (2024): 108783, 10.1016/j.ejso.2024.108783. 39488491

[53] S. Sarkar , X. Zheng , G. C. Clair , et al., “Exploring new Frontiers in Type 1 Diabetes Through Advanced Mass‐Spectrometry‐Based Molecular Measurements,” Trends in Molecular Medicine 30 (2024): 1137–1151, 10.1016/j.molmed.2024.07.009. 39152082 PMC11631641

[54] Z. He , Q. Luo , Z. Liu , and L. Gong , “Extensive Evaluation of Sample Preparation Workflow for Gas Chromatography‐Mass Spectrometry‐Based Plasma Metabolomics and Its Application in Rheumatoid Arthritis,” Analytica Chimica Acta 1131 (2020): 136–145, 10.1016/j.aca.2020.06.029. 32928474

[55] E. N. Ebbel , N. Leymarie , S. Schiavo , et al., “Identification of Phenylbutyrate‐Generated Metabolites in Huntington Disease Patients Using Parallel Liquid Chromatography/Electrochemical Array/Mass Spectrometry and Off‐Line Tandem Mass Spectrometry,” Analytical Biochemistry 399 (2010): 152–161, 10.1016/j.ab.2010.01.010. 20074541 PMC3568495

[56] S. Dagar , M. Sharma , G. Tsaprailis , et al., “Ribosome Profiling and Mass Spectrometry Reveal Widespread Mitochondrial Translation Defects in a Striatal Cell Model of Huntington Disease,” Molecular & Cellular Proteomics 23 (2024): 100746, 10.1016/j.mcpro.2024.100746. 38447791 PMC11040134

[57] M. Zhang , H. Zhang , B. Yan , M. Ren , W. Wang , and T. Zhang , “Diagnostic Performance of Matrix‐Assisted Laser Desorption Ionization Time‐of‐Flight Mass Spectrometry (MALDI‐TOF MS) in Bronchoalveolar Lavage Fluid for Pulmonary Tuberculosis in HIV‐Infected Patients,” Journal of Clinical Tuberculosis and Other Mycobacterial Diseases 36 (2024): 100459, 10.1016/j.jctube.2024.100459. 38983443 PMC11231557

[58] Y. Lu , C. Zhou , R. Yan , et al., “Dynamic Metabolic Profiles for HBeAg Seroconversion in Chronic Hepatitis B (CHB) Patients by Gas Chromatography‐Mass Spectrometry (GC‐MS),” Journal of Pharmaceutical and Biomedical Analysis 206 (2021): 114349, 10.1016/j.jpba.2021.114349. 34597840

[59] R. Uddin and K. M. Downard , “Subtyping of Hepatitis C Virus With High Resolution Mass Spectrometry,” Clinical Mass Spectrometry 4‐5 (2017): 19–24, 10.1016/j.clinms.2017.08.003. PMC11322781 39193130

[60] D. Jain , J. A. Green , S. Bastacky , J. D. Theis , and S. Sethi , “Membranoproliferative Glomerulonephritis: The Role for Laser Microdissection and Mass Spectrometry,” American Journal of Kidney Diseases 63 (2014): 324–328, 10.1053/j.ajkd.2013.09.007. 24145022

[61] N. L. Anderson and N. G. Anderson , “The Human Plasma Proteome,” Molecular & Cellular Proteomics 1 (2002): 845–867, 10.1074/mcp.R200007-MCP200. 12488461

[62] Z. Wang , S. Hill , J. M. Luther , D. L. Hachey , and K. L. Schey , “Proteomic Analysis of Urine Exosomes by Multidimensional Protein Identification Technology (MudPIT),” Proteomics 12 (2012): 329–338, 10.1002/pmic.201100477. 22106071 PMC3517144

[63] D. Dytfeld , M. Luczak , T. Szczepaniak , et al., “Comparative Proteomic Profiling of Sera From Patients With Refractory Multiple Myeloma Reveals Pathways and Biomarkers Predicting Response to Bortezomib‐Based Therapy,” Blood 128 (2016): 2092, 10.1182/blood.V128.22.2092.2092. 28546528

[64] J. D. Sipe , M. D. Benson , J. N. Buxbaum , et al., “Amyloid Fibril Protein Nomenclature: 2012 Recommendations From the Nomenclature Committee of the International Society of Amyloidosis,” Amyloid 19 (2012): 167–170, 10.3109/13506129.2012.734345. 23113696

[65] S. Ihne , C. Morbach , C. Sommer , A. Geier , S. Knop , and S. Störk , “Amyloidosis—The Diagnosis and Treatment of an Underdiagnosed Disease,” Deutsches Ärzteblatt International 117 (2020): 159–166, 10.3238/arztebl.2020.0159. 32295695 PMC7171477

[66] S. V. Rajkumar , “Multiple Myeloma: 2020 Update on Diagnosis, Risk‐Stratification and Management,” American Journal of Hematology 95 (2020): 548–567, 10.1002/ajh.25791. 32212178

[67] O. Landgren , R. A. Kyle , R. M. Pfeiffer , et al., “Monoclonal Gammopathy of Undetermined Significance (MGUS) Consistently Precedes Multiple Myeloma: a Prospective Study,” Blood 113 (2009): 5412–5417, 10.1182/blood-2008-12-194241. 19179464 PMC2689042

[68] A. Nevone , M. Girelli , S. Mangiacavalli , et al., “An N‐Glycosylation Hotspot in Immunoglobulin κ Light Chains Is Associated With AL Amyloidosis,” Leukemia 36 (2022): 2076–2085, 10.1038/s41375-022-01599-w. 35610346

[69] D. Barnidge , D. Troske , S. North , G. Wallis , M. Perkins , and S. Harding , “Endogenous Monoclonal Immunoglobulins Analyzed Using the EXENT® Solution and LC‐MS,” Journal of Mass Spectrometry and Advances in the Clinical Lab 32 (2024): 31–40, 10.1016/j.jmsacl.2024.02.002. 38405412 PMC10891330

[70] K. Li , D. Barnidge , M. Krevvata , M. C. Perkins , R. Verona , and E. Zudaire , “Comparison of the Analytical Performance of EXENT®, a Mass Spectrometry‐Based Assessment of M‐Protein, to SPEP and NGS‐Based MRD in Multiple Myeloma Patient Samples,” Blood 140 (2022): 12446–12447, 10.1182/blood-2022-160249.

[71] O. Berlanga , J. Birtwistle , S. Allen , G. Malin , and G. Lakos , “B‐371 Two Cases of Multiple Myeloma Achieving Complete Remission but Presenting Residual M‐Protein by the EXENT® Solution,” Clinical Chemistry 69 (2023): hvad097.680, 10.1093/clinchem/hvad097.680.

[72] S. Allen , J. Birtwistle , O. Berlanga , G. Malin , and G. Lakos , “B‐180 the EXENT® Solution Provides Evidence for High Prevalence of Multiple M‐Proteins in Monoclonal Gammopathies,” Clinical Chemistry 69 (2023): hvad097.511, 10.1093/clinchem/hvad097.511.

[73] M. Vignon , A. Bastide , A. Attina , et al., “Multiplexed LC‐MS/MS Quantification of Salivary RNA Modifications in Periodontitis,” Journal of Periodontal Research 58 (2023): 959–967, 10.1111/jre.13155. 37349891

[74] N. Foulon , E. Goonatilleke , M. J. MacCoss , M. A. Emrick , and A. N. Hoofnagle , “Multiplexed Quantification of Insulin and C‐Peptide by LC‐MS/MS Without the Use of Antibodies,” Journal of Mass Spectrometry and Advances in the Clinical Lab 25 (2022): 19–26, 10.1016/j.jmsacl.2022.06.003. 35734440 PMC9207678

[75] A. Panigrahi , L. Zhang , J. Benicky , M. Sanda , J. Ahn , and R. Goldman , “A Multiplexed Microflow LC–MS/MS‐PRM Assay for Serologic Quantification of IgG N‐ and HPX O‐ Glycoforms in Liver Fibrosis,” Scientific Reports 13 (2023): 606, 10.1038/s41598-023-27382-0. 36635317 PMC9837073

[76] L. Kang , N. Weng , and W. Jian , “LC–MS Bioanalysis of Intact Proteins and Peptides,” Biomedical Chromatography 34 (2020): e4633, 10.1002/bmc.4633. 31257628

[77] L. Liu , L. Zhang , X. Zheng , X. Liu , W. Liu , and J. Wu , “LC–MS/MS‐Based Multiplex Antibacterial Platform for Therapeutic Drug Monitoring in Intensive Care Unit Patients,” Frontiers in Pharmacology 14 (2023): 1116071, 10.3389/fphar.2023.1116071. 37144212 PMC10151781

[78] M. A. Kuzyk , D. Smith , J. Yang , et al., “Multiple Reaction Monitoring‐Based, Multiplexed, Absolute Quantitation of 45 Proteins in Human Plasma,” Molecular & Cellular Proteomics 8 (2009): 1860–1877, 10.1074/mcp.M800540-MCP200. 19411661 PMC2722777

[79] S. Gallien , E. Duriez , C. Crone , M. Kellmann , T. Moehring , and B. Domon , “Targeted Proteomic Quantification on Quadrupole‐Orbitrap Mass Spectrometer,” Molecular & Cellular Proteomics 11 (2012): 1709–1723, 10.1074/mcp.O112.019802. 22962056 PMC3518128

[80] M. Qu , B. An , S. Shen , et al., “Qualitative and Quantitative Characterization of Protein Biotherapeutics With Liquid Chromatography Mass Spectrometry,” Mass Spectrometry Reviews 36 (2017): 734–754, 10.1002/mas.21500. 27097288

[81] J. G. Meyer , “Fast Proteome Identification and Quantification from Data‐Dependent Acquisition–Tandem Mass Spectrometry (DDA MS/MS) Using Free Software Tools,” Methods and Protocols 2, no. 1 (2019): 8, 10.3390/mps2010008. 31008411 PMC6469856

[82] A. Doerr , “DIA Mass Spectrometry,” Nature Methods 12 (2015): 35, 10.1038/nmeth.3234.

[83] K. Barkovits , A. Linden , S. Galozzi , et al., “Characterization of Cerebrospinal Fluid via Data‐Independent Acquisition Mass Spectrometry,” Journal of Proteome Research 17 (2018): 3418–3430, 10.1021/acs.jproteome.8b00308. 30207155

[84] L. Krasny and P. H. Huang , “Data‐Independent Acquisition Mass Spectrometry (DIA‐MS) for Proteomic Applications in Oncology,” Molecular Omics 17 (2021): 29–42, 10.1039/D0MO00072H. 33034323

[85] M. Iwasaki , R. Nishimura , T. Yamakawa , Y. Miyamoto , T. Tabata , and M. Narita , “Differences in Uniquely Identified Peptides Between ddaPASEF and diaPASEF,” Cells 13 (2024): 1848, 10.3390/cells13221848. 39594597 PMC11592772

[86] C. D. Kelstrup , C. Young , R. Lavallee , M. L. Nielsen , and J. V. Olsen , “Optimized Fast and Sensitive Acquisition Methods for Shotgun Proteomics on a Quadrupole Orbitrap Mass Spectrometer,” Journal of Proteome Research 11 (2012): 3487–3497, 10.1021/pr3000249. 22537090

[87] P. D. Szigetvari , S. Patil , E. Birkeland , R. Kleppe , and J. Haavik , “The Effects of Phenylalanine and Tyrosine Levels on Dopamine Production in rat PC12 Cells. Implications for Treatment of Phenylketonuria, Tyrosinemia Type 1 and Comorbid Neurodevelopmental Disorders,” Neurochemistry International 171 (2023): 105629, 10.1016/j.neuint.2023.105629. 37865339

[88] R. Zong , Y. Liu , M. Zhang , et al., “β‐Catenin Disruption Decreases Macrophage Exosomal α‐SNAP and Impedes Treg Differentiation in Acute Liver Injury,” JCI Insight 10 (2024): e182515, 10.1172/jci.insight.182515. 39560996 PMC11721303

[89] B. Peng , “N‐Glycan Profiling of Medulloblastoma in Patients at Different Proteome Subtypes of Disease,” (2023).

[90] A. V. Cioanca , Y. Wooff , R. Aggio‐Bruce , R. Sekar , C. Dietrich , and R. Natoli , “Multiomic Integration Reveals Neuronal‐Extracellular Vesicle Coordination of Gliotic Responses in Degeneration,” Journal of Extracellular Vesicles 12 (2023): e12393, 10.1002/jev2.12393. 38082562 PMC10714032

[91] M. Al Tarrass , “Décryptage des modifications précoces du phosphoprotéome en réponse à BMP9 et BMP10 dans les cellules endothéliales,” (2023).

[92] J. T. Aguilan , J. Lim , S. Racine‐Brzostek , et al., “Effect of Dynamic Exclusion and the Use of FAIMS, DIA and MALDI‐Mass Spectrometry Imaging With ion Mobility on Amyloid Protein Identification,” Clinical Proteomics 21 (2024): 47, 10.1186/s12014-024-09500-w. 38961380 PMC11223398

[93] A. Hu , W. S. Noble , and A. Wolf‐Yadlin , “Technical Advances in Proteomics: New Developments in Data‐Independent Acquisition,” F1000Res 5 (2016): 419, 10.12688/f1000research.7042.1. PMC4821292 27092249

[94] Y. Jiang , D. DeBord , H. Vitrac , et al., “The Future of Proteomics Is up in the Air: Can Ion Mobility Replace Liquid Chromatography for High Throughput Proteomics?,” Journal of Proteome Research 23 (2024): 1871–1882, 10.1021/acs.jproteome.4c00248. 38713528 PMC11161313

[95] J. McMillen , D. B. Gutierrez , A. M. Judd , J. Spraggins , and R. M. Caprioli , “Tryptic Peptide Signal Enhancement via MALDI IMS Post‐ionization (MALDI‐2) From Thin Tissue Sections,” ChemRxiv, (2020), 10.26434/chemrxiv.13483302.v1.

[96] W. Aftab , S. Lahiri , and A. Imhof , “ImShot: An Open‐Source Software for Probabilistic Identification of Proteins In Situ and Visualization of Proteomics Data,” Molecular & Cellular Proteomics 21 (2022): 100242, 10.1016/j.mcpro.2022.100242. 35569805 PMC9194865

[97] V. R. Umapathy , P. M. Natarajan , and B. Swamikannu , “Review Insights on Salivary Proteomics Biomarkers in Oral Cancer Detection and Diagnosis,” Molecules 28 (2023): 5283, 10.3390/molecules28135283. 37446943 PMC10343386

[98] R. Tomioka , K. Ogata , and Y. Ishihama , “Quantitation of Host Cell Proteins by Capillary LC/IMS/MS/MS in Combination With Rapid Digestion on Immobilized Trypsin Column Under Native Conditions,” Mass Spectrometry 13, no. 1 (2024): A0152–A0152, 10.5702/massspectrometry.A0152. 39296308 PMC11409222

[99] S. Dasari , M. C. Kohlhagen , A. Dispenzieri , et al., “Detection of Plasma Cell Disorders by Mass Spectrometry: A Comprehensive Review of 19,523 Cases,” Mayo Clinic Proceedings 97 (2022): 294–307, 10.1016/j.mayocp.2021.07.024. 34887112

[100] Y. Liang , H. Acor , M. McCown , et al., “Fully Automated Sample Processing and Analysis Workflow for Low‐Input Proteome Profiling,” Analytical Chemistry 93 (2021): 1658–1666, 10.1021/acs.analchem.0c04240. 33352054 PMC8140400

[101] T. Müller , M. Kalxdorf , R. Longuespée , D. N. Kazdal , A. Stenzinger , and J. Krijgsveld , “Automated Sample Preparation With SP 3 for low‐Input Clinical Proteomics,” Molecular Systems Biology 16 (2020): e9111, 10.15252/msb.20199111. 32129943 PMC6966100

[102] C. B. Messner , V. Demichev , D. Wendisch , et al., “Ultra‐High‐Throughput Clinical Proteomics Reveals Classifiers of COVID‐19 Infection,” Cell Systems 11 (2020): 11–24.e4, 10.1016/j.cels.2020.05.012. 32619549 PMC7264033

[103] N. P. M. Smit , L. R. Ruhaak , F. P. H. T. M. Romijn , M. M. Pieterse , Y. E. M. van der Burgt , and C. M. Cobbaert , “The Time Has Come for Quantitative Protein Mass Spectrometry Tests That Target Unmet Clinical Needs,” Journal of the American Society for Mass Spectrometry 32 (2021): 636–647, 10.1021/jasms.0c00379. 33522792 PMC7944566

[104] D. N. Perkins , D. J. C. Pappin , D. M. Creasy , and J. S. Cottrell , “Probability‐Based Protein Identification by Searching Sequence Databases Using Mass Spectrometry Data,” Electrophoresis 20 (1999): 3551–3567, 10.1002/(SICI)1522-2683(19991201)20:18<3551::AID-ELPS3551>3.0.CO;2-2. 10612281

[105] J. K. Eng , A. L. McCormack , and J. R. Yates , “An Approach to Correlate Tandem Mass Spectral Data of Peptides With Amino Acid Sequences in a Protein Database,” Journal of the American Society for Mass Spectrometry 5 (1994): 976–989, 10.1016/1044-0305(94)80016-2. 24226387

[106] R. Craig and R. C. Beavis , “TANDEM: Matching Proteins With Tandem Mass Spectra,” Bioinformatics 20 (2004): 1466–1467, 10.1093/bioinformatics/bth092. 14976030

[107] B. Ma , K. Zhang , C. Hendrie , et al., “PEAKS: Powerful Software for Peptide De Novo Sequencing by Tandem Mass Spectrometry,” Rapid Communications in Mass Spectrometry 17 (2003): 2337–2342, 10.1002/rcm.1196. 14558135

[108] A. Frank and P. Pevzner , “PepNovo: De Novo Peptide Sequencing via Probabilistic Network Modeling,” Analytical Chemistry 77 (2005): 964–973, 10.1021/ac048788h. 15858974

[109] B. Fischer , V. Roth , F. Roos , et al., “NovoHMM: A Hidden Markov Model for De Novo Peptide Sequencing,” Analytical Chemistry 77 (2005): 7265–7273, 10.1021/ac0508853. 16285674

[110] R. Aebersold and M. Mann , “Mass Spectrometry‐Based Proteomics,” Nature 422 (2003): 198–207, 10.1038/nature01511. 12634793

[111] B. Domon and R. Aebersold , “Mass Spectrometry and Protein Analysis,” Science 312 (2006): 212–217, 10.1126/science.1124619. 16614208

[112] C. Chen , J. Hou , J. J. Tanner , and J. Cheng , “Bioinformatics Methods for Mass Spectrometry‐Based Proteomics Data Analysis,” International Journal of Molecular Sciences 21 (2020): 2873, 10.3390/ijms21082873. 32326049 PMC7216093

[113] The UniProt Consortium , “UniProt: a Worldwide Hub of Protein Knowledge,” Nucleic Acids Research 47 (2019): D506–D515, 10.1093/nar/gky1049. 30395287 PMC6323992

[114] J. E. Elias , W. Haas , B. K. Faherty , and S. P. Gygi , “Comparative Evaluation of Mass Spectrometry Platforms Used in Large‐Scale Proteomics Investigations,” Nature Methods 2 (2005): 667–675, 10.1038/nmeth785. 16118637

[115] J. Colinge , A. Masselot , M. Giron , T. Dessingy , and J. Magnin , “OLAV: Towards High‐Throughput Tandem Mass Spectrometry Data Identification,” Proteomics 3 (2003): 1454–1463, 10.1002/pmic.200300485. 12923771

[116] E. A. Kapp , F. Schütz , L. M. Connolly , et al., “An Evaluation, Comparison, and Accurate Benchmarking of Several Publicly Available MS/MS Search Algorithms: Sensitivity and Specificity Analysis,” Proteomics 5 (2005): 3475–3490, 10.1002/pmic.200500126. 16047398

[117] H. Choi and A. I. Nesvizhskii , “False Discovery Rates and Related Statistical Concepts in Mass Spectrometry‐Based Proteomics,” Journal of Proteome Research 7 (2008): 47–50, 10.1021/pr700747q. 18067251

[118] J. S. Jeong , L. Jiang , E. Albino , et al., “Rapid Identification of Monospecific Monoclonal Antibodies Using a Human Proteome Microarray,” Molecular & Cellular Proteomics 11 (2012): O111.016253, 10.1074/mcp.O111.016253. PMC3433917 22307071

[119] S. McIlwain , K. Tamura , A. Kertesz‐Farkas , et al., “Crux: Rapid Open Source Protein Tandem Mass Spectrometry Analysis,” Journal of Proteome Research 13 (2014): 4488–4491, 10.1021/pr500741y. 25182276 PMC4184452

[120] P. Hernandez , M. Müller , and R. D. Appel , “Automated Protein Identification by Tandem Mass Spectrometry: Issues and Strategies,” Mass Spectrometry Reviews 25 (2006): 235–254, 10.1002/mas.20068. 16284939

[121] J. Cox and M. Mann , “MaxQuant Enables High Peptide Identification Rates, Individualized p.p.b.‐Range Mass Accuracies and Proteome‐Wide Protein Quantification,” Nature Biotechnology 26 (2008): 1367–1372, 10.1038/nbt.1511. 19029910

[122] S. Sethi , J. D. Theis , N. Leung , et al., “Mass Spectrometry–Based Proteomic Diagnosis of Renal Immunoglobulin Heavy Chain Amyloidosis,” Clinical Journal of the American Society of Nephrology 5, no. 12 (2010): 2180–2187, 10.2215/CJN.02890310. 20876678 PMC2994078

